# Cellular interferon-gamma and interleukin-2 responses to SARS-CoV-2 structural proteins are broader and higher in those vaccinated after SARS-CoV-2 infection compared to vaccinees without prior SARS-CoV-2 infection

**DOI:** 10.1371/journal.pone.0276241

**Published:** 2022-10-17

**Authors:** Martha Sedegah, Chad Porter, Emilie Goguet, Harini Ganeshan, Maria Belmonte, Jun Huang, Arnel Belmonte, Sandra Inoue, Neda Acheampong, Allison M. W. Malloy, Monique Hollis-Perry, Belinda Jackson-Thompson, Kathy F. Ramsey, Yolanda Alcorta, Santina E. Maiolatesi, Gregory Wang, Anatolio E. Reyes, Luca Illinik, Margaret Sanchez-Edwards, Timothy H. Burgess, Christopher C. Broder, Eric D. Laing, Simon D. Pollett, Eileen Villasante, Edward Mitre, Michael R. Hollingdale

**Affiliations:** 1 Agile Vaccines and Therapeutics, Naval Medical Research Center, Silver Spring, MD, United States of America; 2 Translational Clinical Research Department, Naval Medical Research Center, Silver Spring, MD, United States of America; 3 Department of Microbiology and Immunology, Uniformed Services University of the Health Sciences, Bethesda, MD, United States of America; 4 Henry M. Jackson Foundation for the Advancement of Military Medicine, Inc., Bethesda, MD, United States of America; 5 General Dynamics Information Technology, Falls Church, VA, United States of America; 6 Department of Pediatrics, Uniformed Services University of the Health Sciences, Bethesda, MD, United States of America; 7 Clinical Trials Center, Naval Medical Research Center, Silver Spring, MD, United States of America; 8 Infectious Diseases Clinical Research Program, Department of Preventive Medicine and Biostatistics, Uniformed Services University of the Health Sciences, Bethesda, MD, United States of America; Translational Health Science & Technology Institute, INDIA

## Abstract

Class I- and Class II-restricted epitopes have been identified across the SARS-CoV-2 structural proteome. Vaccine-induced and post-infection SARS-CoV-2 T-cell responses are associated with COVID-19 recovery and protection, but the precise role of T-cell responses remains unclear, and how post-infection vaccination (‘hybrid immunity’) further augments this immunity To accomplish these goals, we studied healthy adult healthcare workers who were (a) uninfected and unvaccinated (n = 12), (b) uninfected and vaccinated with Pfizer-BioNTech BNT162b2 vaccine (2 doses n = 177, one dose n = 1) or Moderna mRNA-1273 vaccine (one dose, n = 1), and (c) previously infected with SARS-CoV-2 and vaccinated (BNT162b2, two doses, n = 6, one dose n = 1; mRNA-1273 two doses, n = 1). Infection status was determined by repeated PCR testing of participants. We used FluoroSpot Interferon-gamma (IFN-γ) and Interleukin-2 (IL-2) assays, using subpools of 15-mer peptides covering the S (10 subpools), N (4 subpools) and M (2 subpools) proteins. Responses were expressed as frequencies (percent positive responders) and magnitudes (spot forming cells/10^6^ cytokine-producing peripheral blood mononuclear cells [PBMCs]). Almost all vaccinated participants with no prior infection exhibited IFN-γ, IL-2 and IFN-γ+IL2 responses to S glycoprotein subpools (89%, 93% and 27%, respectively) mainly directed to the S2 subunit and were more robust than responses to the N or M subpools. However, in previously infected and vaccinated participants IFN-γ, IL-2 and IFN-γ+IL2 responses to S subpools (100%, 100%, 88%) were substantially higher than vaccinated participants with no prior infection and were broader and directed against nine of the 10 S glycoprotein subpools spanning the S1 and S2 subunits, and all the N and M subpools. 50% of uninfected and unvaccinated individuals had IFN-γ but not IL2 or IFN-γ+IL2 responses against one S and one M subpools that were not increased after vaccination of uninfected or SARS-CoV-2-infected participants. Summed IFN-γ, IL-2, and IFN-γ+IL2 responses to S correlated with IgG responses to the S glycoprotein. These studies demonstrated that vaccinations with BNT162b2 or mRNA-1273 results in T cell-specific responses primarily against epitopes in the S2 subunit of the S glycoprotein, and that individuals that are vaccinated after SARS-CoV-2 infection develop broader and greater T cell responses to S1 and S2 subunits as well as the N and M proteins.

## Introduction

Coronavirus disease 2019 (COVID-19) is caused by the severe acute respiratory syndrome coronavirus 2 (SARS-CoV-2) infection [1], and is responsible for more than 605 million confirmed cases, and more than 6.5 million deaths as of September 12, 2022 (COVID-19 report of the World Health Organization). The emergence of the SARS-CoV-2 variant Omicron [2] has underscored the need to understand the breadth and depth of immunity conferred by prior infection and vaccination.

COVID-19 vaccines inducing neutralizing antibodies to the spike (S) glycoprotein have received U.S. Food and Drug Administration (FDA) approval and are in widespread use. Most research on vaccine correlates of protection has focused on such antibody responses [3]. However, T cell immunity has been shown to be a key determinant of COVID-19 protection after either vaccination or infection, and the persistence of SARS-CoV-2-specific T cell responses is also an important consideration in vaccine evaluation and development [4–7]. T cell responses to SARS-CoV-2 recognize epitopes in structural proteins such as the spike (S), nucleocapsid (N), membrane (M), and small envelope (E) proteins, as well as other nonstructural proteins [8, 9]. Identification of antigen-specific T cell responses could support the inclusion of antigenic vaccine targets in addition to the S glycoprotein in next generation COVID-19 vaccines.

Recent studies have demonstrated that prior SARS-CoV-2 infection of individuals and vaccination may have greater protection against clinical disease from SARS-CoV-2 infection than that conferred by either prior infection or vaccination alone [10, 11]. While hybrid immunity has been shown to be correlated with broader and more robust humoral immune responses, there is less known about how prior infection shapes the breadth and magnitude of T-cell immunity. We used peripheral blood mononuclear cell (PBMC) samples from participants in the Prospective Assessment of SARS-CoV-2 Seroconversion (PASS) study, an observational cohort study of SARS-CoV-2 infection and vaccination in at-risk health care workers [12]. All vaccinated participants seroconverted of IgG antibodies to the Spike S protein that waned at 6 months post-vaccination [13]. In this study, we sought to characterize the influence of prior COVID-19 infection on the breadth and magnitude of T cell responses after 2 doses of the mRNA COVID-19 vaccines by comparing these responses to individuals that had been vaccinated with no prior SARS-CoV-2 infection, or with neither prior infection nor vaccination.

We used FluoroSpot IFN-γ, IL-2 and IFN-γ+IL2 assays to identify T cell immunodominant regions and sequences within the S, N and M proteins recognized by vaccination with current SARS-CoV-2 vaccines of participants without or with prior SARS-CoV-2 infection We then compared these responses with previously reported IgG responses to the S protein [13] to determine whether both may have played a role in protective responses. Our ultimate aim is to identify T cell epitopes within these immunodominant regions that are conserved across SARS-CoV, SARS-CoV-2, and common cold HCoVs for the potential development of potent biomarkers of protective immunity and as future targets of a multi-epitope vaccine that would be broadly effective against these and future pandemic-causing HCoVs.

## Methods

### Ethics

The Prospective Assessment of SARS-CoV-2 Seroconversion (PASS) study was initiated in August 2020 by enrolling 271 SARS-CoV-2 seronegative healthcare workers (HCWs) recruited from Walter Reed National Military Medical Center (WRNMMC), with no history of COVID-19, over a six-month time-period. The study protocol was approved by the Uniformed Services University of the Health Sciences (USUHS) Institutional Review Board (FWA 00001628; DoD Assurance P60001) and the NMRC Institutional Review Board (IRB) in compliance with all applicable US federal regulations governing the protection of human participants. NMRC holds a Federalwide Assurance from the Office of Human Research Protections (OHRP) under the Department of Health and Human Services, and a Department of Defense/Department of the Navy Assurance for human subject protections. All key personnel were certified as having completed mandatory human participants’ protection curricula and training under the direction of the WRAIR IRB and Human Participants Protections Branch (HSPB) or the NMRC IRB and Office of Research Administration (ORA). All participants provided written informed consent.

### Study participants

PBMCs used for this study were obtained from participants in the PASS Study [12]. Prior to enrollment, COVID-19 diagnosis by qPCR test or positive SARS-CoV-2 Ig serology were exclusionary. Throughout the study period, participants were evaluated for evidence of SARS-CoV-2 infection by monthly testing for IgG antibodies against SARS-CoV-2 spike glycoprotein as well as testing for active SARS-CoV-2 infection by nasopharyngeal swab PCR assay whenever they experienced symptoms of a viral respiratory infection. Of the 271 individuals enrolled in the study between August of 2020 and March of 2021, 199 agreed to undergo an additional blood draw for T-cell epitope analyses between March and June of 2021. PASS is projected to continue to 2024 [12].

### Samples

PBMCs for measuring cell-mediated immunity by FluoroSpot assay were collected from Group 1, Group 2, and Group 3 (as defined in Results) participants between April and June of 2021, approximately 121 days after the last vaccination (Group 2: Mean 121.38 days, St.D. 13.55 days; Group 3: Mean 109.13 days, St.D. 15.07 days), isolated from heparin tubes within 24 hours, and stored in liquid nitrogen (10-20x10^6^ cells/mL) until used. Cryopreserved PBMCs were thawed, washed, counted, and used in the FluoroSpot assay to quantify the number of cells secreting either IFN-γ or Interleukin-2 (IL2), as previously reported [14–16].

### Peptide subpools and megapools

**Fig 1:**
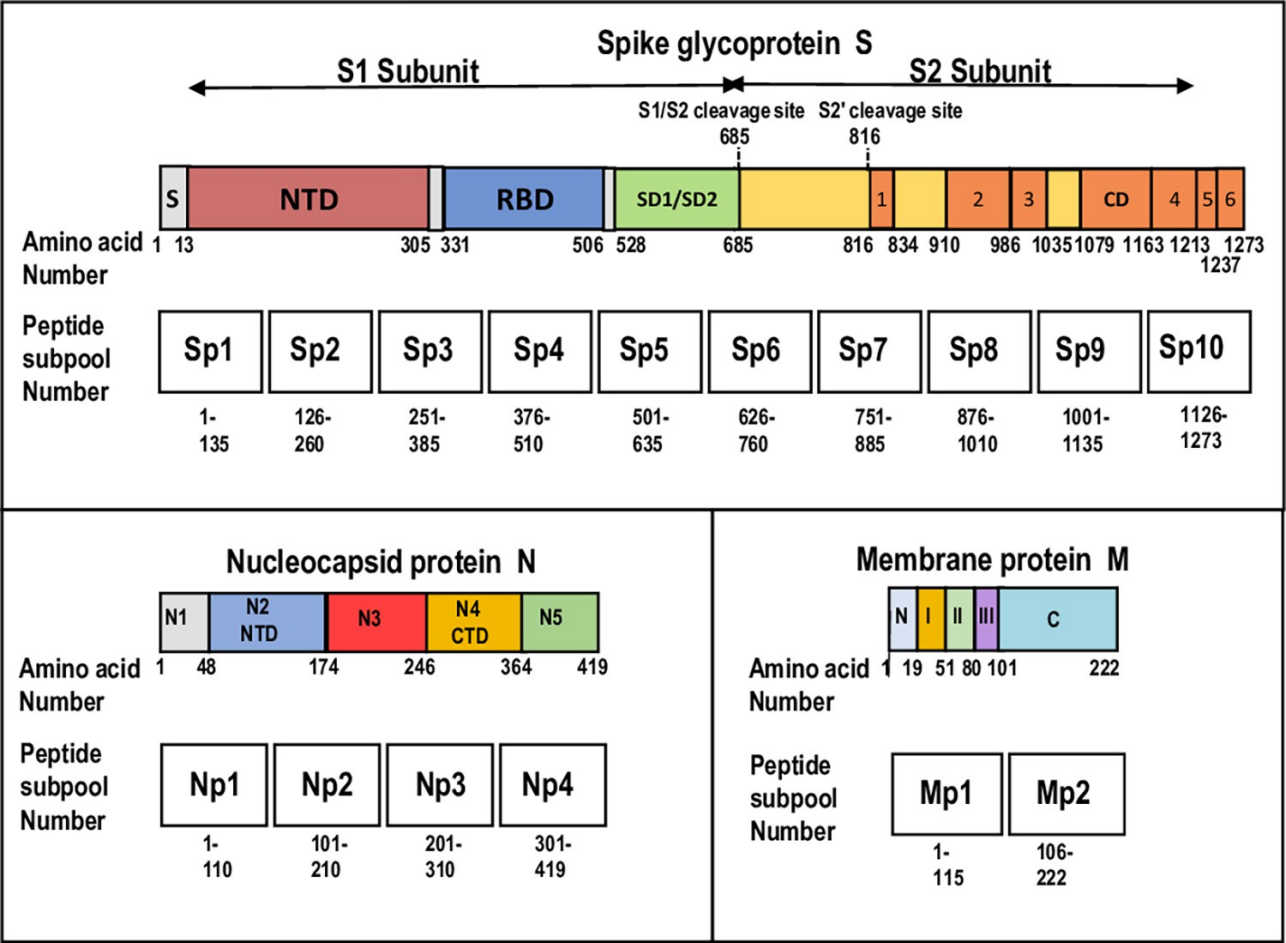
S, N, and M peptide subpools and their relationship to each protein structure. The structures of the S glycoprotein, and N and M proteins and their individual domains are aligned with the peptide subpools for each protein. Since constituent 15mer peptides partially overlap, each subpool also partially overlaps adjoining subpools. Consensus amino acid positions are shown in each protein. Some differences are reported, for example the S heptad repeat is variously reported to end at amino acid 1211 (reference #19), and 1213 (reference #20). **Spike S glycoprotein** (reference #20): Signal peptide (S); N-terminal domain (NTD); RBD (receptor-binding domain); SD1/SD2 (subdomain 1 and 2); 1 (fusion peptide); 2 (HR1, heptad repeat); 3 (central helix); CD (connector domain); 4 (HR2, heptad repeat); 5 (transmembrane domain); 6 (cytoplasmic tail); the S1/S2 and S2’ cleavage sites (reference #23). S protein subpools predominantly aligned with S domains: Sp1, Sp2 NTD; Sp3 NTD/RBD; Sp4 RBD; Sp5 SD1/SD2; Sp6 SD1/SD2 including S1/S2 cleavage site; Sp7 fusion peptide; Sp8 heptad repeat; Sp9 central helix/connector domain; Sp10 heptad repeat/transmembrane domain/cytoplasmic tail. **Nucleocapsid protein N** (reference #24): domain N1; domain N2 (N-terminal domain [NTD] or RNA binding domain); domain N3 (contains a serine-arginine rich region); domain N4 (C-terminal domain [CTD]; domain N5. N protein subpools predominantly aligned with N domains: Np1 domains N1 and part of N2; Np2 domains N2 and N3; Np3 domains N3 and N4; Np4 domains N4 and N5. **Membrane protein M** (reference #26): N (NTD, N-terminal domain); I, II and III (transmembrane domains); C (CTD, C-terminal domain). M protein subpools predominantly aligned with M domains: Mp1 NTD, 1, II and III; Mp2 CTD.

All peptides were obtained from Mimotopes (Melbourne, Australia) and were based on the USA-WA1/2020 strain of SARS-CoV-2 (GenPept. QH060594 [17]. The full-length spike glycoprotein (S) [18–22], full length nucleocapsid (N) protein [23, 24], and the full-length membrane (M) protein [25], were each covered by a series of 15mer (aa) peptides overlapping by 5 amino acids. Protein-specific subpools were designed to have consistent numbers of 20–27 15mers (Fig 1): S glycoprotein subpools Sp1 –Sp9 each contained 25 15mer peptides, and Sp10 contained the remaining 27 15mers, and the S megapool, Smp, contained all 15mer peptides spanning S glycoprotein; N protein subpools Np1 –Np3 each contained 20 15mers, and Np4 contained the remaining 22 15mers, and the N megapool, Nmp, contained all 15mer peptides spanning N protein; M protein subpools Mp1 and Mp2 contained 21 and 22 15mers, respectively, and the M megapool, Mmp, contained all 15mer peptides spanning M protein. Since the 15mer constituent peptides overlapped each other, individual subpools also overlapped adjacent subpools. Each subpool can be partly aligned with the structural domains of each protein [19, 23, 25], although many subpools overlap more than one domain and were not designed to be domain-specific.

### FluoroSpot IFN-γ/IL2 assay

Antigen-specific circulating PBMCs were evaluated using pre-coated FluoroSpot plates and kits purchased from Mabtech (Mabtech AB, Nacka Strand, Sweden) and used according to the manufacturer’s instructions. The *ex vivo* ELISpot and the FluoroSpot assays have been previously described [26–28]. The positive control was CEF-Class I Peptide Pool *Plus* (CTL, Ohio, USA) consisting of 32 peptides corresponding to defined HLA class I-restricted T-cell epitopes from cytomegalovirus, Epstein-Barr virus, and influenza virus [14]. After subtraction of the number of spot-forming cells (sfcs) in negative control wells (no antigen), the net sfcs of the test sample was expressed as sfcs/million (m) PBMCs (sfc/m). The response of each subject to each individual peptide subpool was considered positive when there was at least a doubling of sfc/m in test compared to control wells, and a difference of at least 10 sfc between test and control wells [29]. The summed responses of individual participants against the test proteins were considered positive when there was a positive response to at least one protein-specific peptide subpool. A representative example of spot-forming pattern of responses after stimulation of PBMCs from subjects in our three study groups with one antigen-specific subpool is shown in S1 Fig in S1 File. The magnitudes of responses to S, N, and M subpools were expressed as sfc/m for each protein, and frequencies of responses were the number of participants with a positive response expressed as per cent of the total numbers of participants. For each protein, the value of magnitudes of responses to all subpools (positive and negative) were also added together and expressed as summed magnitude sfc/m for that protein.

Since PBMCs were collected at varying times after the final vaccinations, we compared FluoroSpot IFN-γ, IL2 and IFN-γ+IL2 responses to peptide subpools with times post-vaccination. There was no correlation between these two parameters using regression analyses (S2 Fig in S1 File), confirming that FluoroSpot responses of these PBMCs were dependent only on the stimulating peptide and not time of collection.

### Serum IgG antibody responses to the Spike glycoprotein

Monthly serum samples were screened for IgG against SARS-CoV-2 spike glycoprotein in multiplex microsphere-based immunoassays, as previously described [13].

### Statistical analysis

We measured the magnitudes and frequencies of IFN-γ, IL2, and IFN-γ+IL2 responses to antigen-specific subpools containing 15mer peptides spanning the S, N, and M proteins, and each protein megapool, and compared these antigen-specific responses among the three groups of participants. Initial omnibus null hypotheses compared the magnitude (Kruskal-Wallis) and frequency (Fisher’s Exact) of responses of all groups to each of the subpools, and summed responses to all protein-specific subpools, and were interpreted using a two-sided alpha = 0.05. When rejected, pairwise comparisons were performed and interpreted using a Bonferroni-adjusted alpha = 0.0167. Heatmaps of the Pearson’s correlation across all immune parameters were developed by study group. Principal Component Analysis (PCA) was used to reduce immune response data dimensionality and redundancy, and principal components were analyzed by participants who were infected and vaccinated, those who were uninfected and vaccinated and those who were uninfected and unvaccinated. PCA sampling adequacy was assessed with a Kaiser-Meyer-Olkin (KMO) ≥0.6 required and a significant (p<0.05) Bartlett’s test of sphericity required [30]. All analyses were performed using SAS v.9.4 or JMP v.12.2 (Cary, NC). Correlation between serum IgG antibody Spike glycoprotein and summed IFN-γ, IL2, and IFN-γ+IL2 responses to Spike glycoprotein subpools were analyzed using a non-parametric Spearman correlation, 2-tailed, Confidence Interval 95%, in GraphPad Prism 9.0.2.

## Results

### Study design: Participant groups

At enrollment, all study participants were negative for COVID-19 diagnosis using qPCR tests whenever participants had any symptoms of SARS-CoV-2 infection and by longitudinal monitoring of anti-spike protein IgG serology [12], although we recognize it remains possible that potential prior exposure cannot be completely ruled out despite negative qPCR or seronegativity [31]. Study participants were classified into three groups:

Group 1 (n = 12) participants were not SARS-CoV-2 infected nor vaccinated.

Group 2 (n = 179) participants had no documented SARS-CoV-2 infection by qPCR but had received either the Pfizer-BioNTech BBNT162b2 COVID-19 vaccine [32] (full two dose series: n = 177, approximately 21 days apart [Mean 21.83 days, Standard deviation 3.17 days]; one dose n = 1); or the Moderna mRNA-1273 vaccine [33] (single dose: n = 1).

Group 3 (n = 8 participants) were infected with SARS-CoV-2 that was confirmed by qPCR between 14 and 43 days before vaccination and received either the Pfizer-BioNTech vaccine (full two dose series: n = 6 participants approximately 21 days apart [Mean 23.5 days, St.D. 4.43 days], or single dose: n = 1), or the Moderna vaccine (full two dose series n = 1, 28 days apart); all participants in groups 2 and 3 developed positive IgG antibodies to SARS-CoV-2 S-2P glycoprotein ectodomain trimers [34].

### Summed IFN-γ, IL2, and IFN-γ+IL2 responses to S, N, and M proteins

#### Group 1: Uninfected and unvaccinated participants (Table 1)

The frequencies and magnitudes of summed IFN-γ responses to the S (67%, 48 sfc/m) and M (42%, 22 sfc/m) subpools were higher than the N subpools (8%, 1 sfc/m). The frequency of summed IL2 responses to the S, M and N subpools were rare (≤8%), and magnitudes of IL2 responses the S (42 sfc/m) and N (10 sfc/m) subpools represent single positive participants. IFN-γ+IL2 responses were largely absent to the S, N and M proteins.

**Table 1:** Summed FluoroSpot IFN-γ, IL2, and IFN-γ+IL2 responses to S, N and M proteins.

Group		#		IFN-γ			IL2			IFN-γ+IL2		
				S	N	M	S	N	M	S	N	M
**1**	**Uninfected & unvaccinated**	**12**	**Median Q1-Q3**	48 35–121	1 0–18	22 11–41	42 25–76	10 4–29	3 0–5	0 0–5	0 0–0	0 0–0
			**# (%) Pos**	8 (67)	1 (8)	5 (42)	1 (8)	1 (8)	0 (0)	1 (8)	0 (0)	0 (0)
**2**	**Uninfected &vaccinated**	**179**	**Median Q1-Q3**	195* 133–308	20* 8–45	25 14–53	246* 148–393	33* 17–57	3 0–10	40* 18–73	3* 0–10	0 0–0
			**# (%) Pos**	159 (89)	49 (27)	93 (52)	166 (93)	65 (36)	1 (0.6)	49 (27)	5 (3)	0 (0)
**3**	**Infected & vaccinated**	**8**	**Median Q1-Q3**	578^ 391–808	189^ 80–230	168^ 86–250	490^ 344–550	130^ 96–252	171^ 54–268	110^ 113–155	34^ 21–69	54^ 28–74
			**# (%) Pos**	8 (100)	7 (88)	8 (100)	8 (100)	6 (75)	7 (88)	7 (88)	4 (50)	5 (63)

The sum of magnitudes of IFN-γ, IL2 and IFN-γ+IL2 responses to S, N and M individual peptide subpools are expressed as the median, and first and third quartiles (Q1 and Q3, respectively) responses (spot forming cells/million PBMC, sfc/m), and frequencies are expressed as the number (#) and % of the participants that were positive (as defined in Methods).

Initial omnibus null hypotheses comparing the summed median magnitude (Kruskal-Wallis) to all pools of each protein were interpreted using an alpha = 0.05. When rejected, pairwise comparisons were performed and interpreted using a Bonferroni-adjusted alpha = 0.0167. *Significant differences medians in Group 2 compared to Group 1

^Significant differences in medians in Group 3 compared to Group 2. Analysis of the frequency of response (Fisher’s Exact) not done.

#### Group 2: Uninfected and vaccinated participants (Table 1)

The frequencies of IFN-γ, IL2 and IFN-γ+IL2 responses to the S (89%, 93%, 27%), N (27%, 36%, 3%), but not M, were higher than Group 1. The magnitudes of IFN-γ, IL2 and IFN-γ+IL2 responses to the S (195 sfc/m, 246 sfc/m, 40 sfc/m) and N (20 sfc/m, 33 sfc/m, 3 sfc/m) subpools, and but not to M subpools, were significantly higher than Group 1.

#### Group 3: Infected and vaccinated participants (Table 1)

The frequencies of summed IFN-γ, IL2 and IFN-γ+IL2 responses to S (100%, 100%, 88%), N (88%, 75%, 50%) and M (100%, 88%, 63%) subpools were all higher than Group 2. The magnitudes of IFN-γ, IL2 and IFN-γ+IL2 responses to S (578 sfc/m, 490 sfc/m, 110 sfc/m), N (189 sfc/m, 130 sfc/m, 34 sfc/m) and M (168 sfc/m, 171 sfc/m, 54 sfc/m) were each significantly higher than Group 2.

#### Interpretation

Since uninfected and unvaccinated participants were negative by PCR and seronegative, we suggest that IFN-γ and IL2 responses to the S and M proteins may represent prior HCoV non-SARS-CoV-2 infections that were not detected by those PCR assays. Vaccination with the S-based vaccine of participants with no prior SARS-CoV-2 infection elicited, as expected, IFN-γ, IL2, and IFN-γ+IL2 responses to the S glycoprotein. However, we also detected IFN-γ, IL2, and IFN-γ+IL2 responses to the N protein (**Table 1**, Group 2). The frequency of IFN-γ responses to the N protein (27%) was similar to the previously reported frequency (26%) of seroconversion to the N protein in this cohort [13]. IFN-γ, IL2, and IFN-γ+IL2 responses to the M protein were similar in uninfected vaccinated and unvaccinated participants. After vaccination of participants with prior SARS-CoV-2 infection, IFN-γ, IL2, and IFN-γ+IL2 responses to the S, N and M proteins were significantly higher than vaccinated non-infected participants. Others have also suggested that vaccination may have resulted in moderate non-specific activation of memory T cells with specificity for cross-reactive sequences from prior HCoV infections [35].

**Fig 2:**
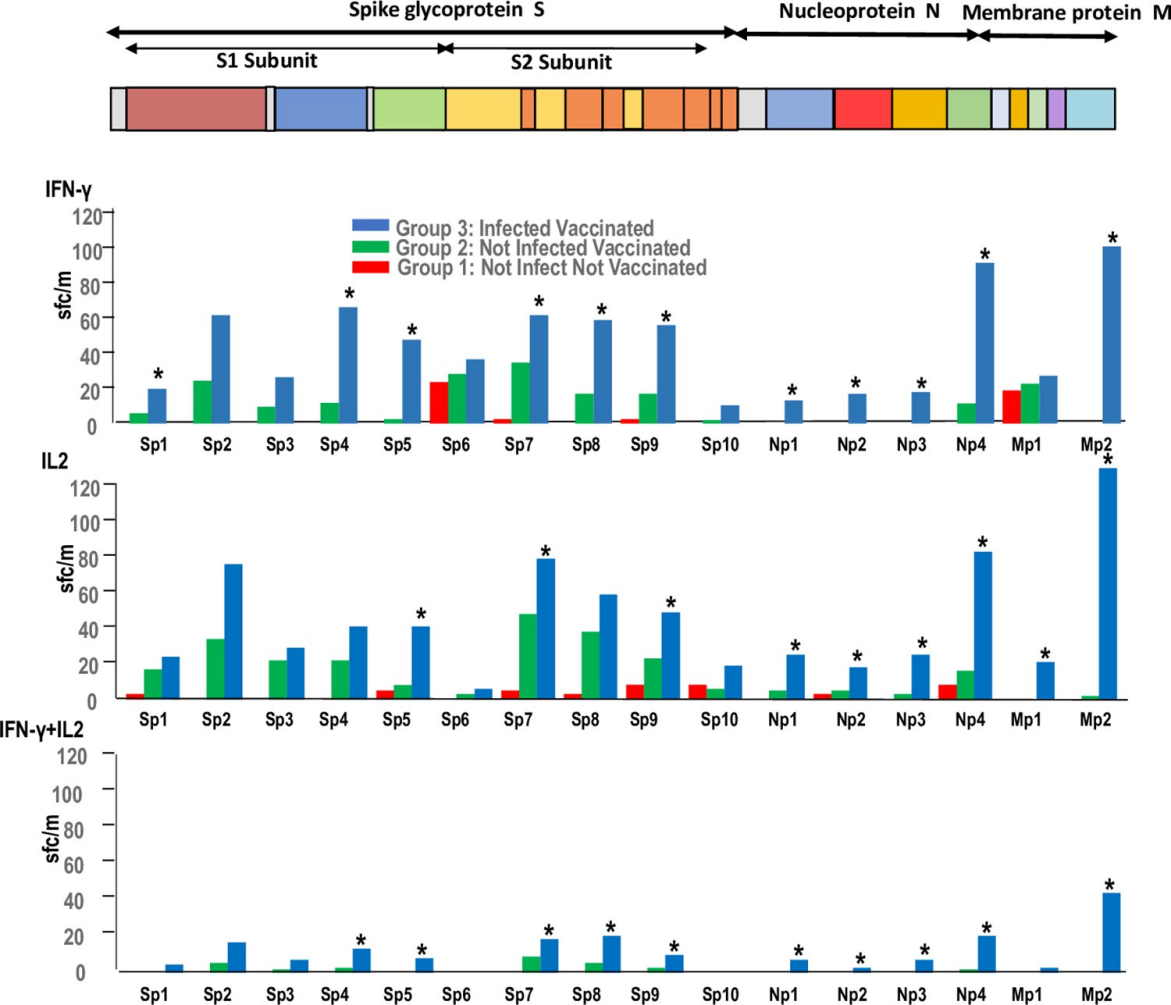
Group 1, Group 2, and Group 3: Median IFN-γ, IL2, and IFN-γ+IL2 responses to S, N, and M subpools. Medians of the magnitudes of responses of Group 1 (uninfected, unvaccinated), Group 2 (uninfected, vaccinated), and Group 3 (infected, vaccinated) IFN-γ, IL2, and IFN-γ+IL2 responses (spot forming cells/million PBMCs, sfc/m) to S glycoprotein (Sp1 –Sp10), N protein (Np1 –Np4), and M protein (Mp1, Mp2) peptide subpools. *Significant differences between Group 2 and Group 3 indicate that prior SARS-CoV-2 infection significantly increased responses to each subpool as indicated.

We next examined IFN-γ, IL2, and IFN-γ+IL2 responses to the individual 10 S, 4 N, and 2 M peptide subpools in each Group (Tables 2–4, and Fig 2) to identify specific recognition of subpools in the three groups.

**Table 2:** FluoroSpot IFN-γ responses to individual S, N, and M peptide subpools.

	IFN-γ							
	GROUP 1 N = 12		GROUP 2 N = 179		GROUP 3 N = 8		KW	Chi-Sq
Peptide Pool	Median Q1-Q3	# Pos (%)	Median Q1-Q3	# Pos (%)	Median Q1-Q3	# Pos (%)	P Mag	P Fre
**Sp1**	0 0–4	0 (0)	6* 2–16	25 (14)	21^ 11–40	3 (38)	**	
**Sp2**	0 0–3	0 (0)	26* 13–44	96 (54)*	66 41–110	7 (88)	**	**
**Sp3**	0 0–0	0 (0)	10* 3–23	43 (24)	28 15–56	4 (50)	**	
**Sp4**	0 0–4	0 (0)	13* 3–24	43 (24)	71^ 40–76	6 (75)^	**	**
**Sp5**	0 0–0	0 (0)	3* 0–13	18 (10)	51 18–89	6 (75)^	**	**
**Sp6**	28 15–46	6 (50)	30 15–54	114 (64)	39 18–58	5 (63)		
**Sp7**	3 1–5	0 (0)	37* 18–60	114 (64)*	66^ 54–325	8 (100)	**	**
**Sp8**	0 0–1	1 (8)	18* 10–37	68 (38)	63^ 40–113	7 (88)^	**	**
**Sp9**	1 0–19	3 (25)	18* 6–38	73 (41)	60^ 39–138	7 (88)	**	
**Sp10**	0 0–5	1 (8)	2 0–8	16 (9)	11 3–26	2 (25)		
**Np1**	0 0–0	0 (0)	0 0–4	9 (5)	14^ 5–50	3 (38)^	**	**
**Np2**	0 0–5	1 (8)	3 0–10	13 (7)	18^ 5–45	3 (38)	**	
**Np3**	0 0–0	0 (0)	0 0–5	6 (3)	19^ 5–49~	3 (38)^	**	**
**Np4**	0 0–10	0 (0)	12* 4–20	39 (22)	98^ 45–136	7 (88)^	**	**
**Mp1**	22 11–39	5 (38)	24 12–43	92 (52)	29 28–48	7 (88)		
**Mp2**	0 0–0	1 (8)	0 0–4	14 (8)	108^ 54–223	8 (100)^	**	**

IFN-γ responses to individual peptide subpools for the S, N and P proteins were expressed as median and range of responses (spot forming cells/million PBMC, sfc/m), and number and % participants that were positive.

Initial omnibus null hypotheses comparing the magnitude (Kruskal-Wallis) and frequency (Fisher’s Exact) of responses of all groups to each of the subpools were interpreted using an alpha = 0.05. When rejected, pairwise comparisons were performed and interpreted using a Bonferroni-adjusted alpha = 0.0167.

*Significant differences between Group 2 compared to Group 1

**^**Significant differences between Group 3 compared to Group 2.

In addition, the magnitudes, and frequencies of responses of each Group were also compared by Kruskall-Wallis (KW) or Chi-Square (Chi-Sq).

**Significant p values indicate that there were significant differences between each group.

**Table 3:** FluoroSpot IL2 responses to individual S, N, and M peptide subpools.

	IL2							
	GROUP 1 N = 12		GROUP 2 N = 179		GROUP 3 N = 8		KW	Chi-Sq
Peptide Pool	Median Q1-Q3	# Pos (%)	Median Q1-Q3	# Pos (%)	Median Q1-Q3	# Pos (%)	P Mag	P Fre
**Sp1**	3 0–8	0 (0)	17* 7–30	59 (33)*	24 15–34	4 (50)	**	
**Sp2**	0 0–3	0 (0)	34* 23–66	123 (69)*	76 43–116	7 (88)	**	**
**Sp3**	0 0–5	0 (0)	22* 8–43	77 (43)*	29 23–48	4 (50)	**	
**Sp4**	0 0–3	0 (0)	22* 0–235	85 (47)*	41 29–68	6 (75)	**	**
**Sp5**	4 0–9	0 (0)	8 10–40	35 (20)	41^ 19–48	5 (63)^	**	**
**Sp6**	0 0–0	0 (0)	3* 0–10	9 (5)	6 0–18	1 (13)		
**Sp7**	2 0–13	0 (0)	48* 28–82	145 (81)*	70^ 64–168	8 (100)	**	**
**Sp8**	3 0–8	0 (0)	38* 19–60	120 (67)*	59 40–84	8 (100)	**	**
**Sp9**	6 2–11	0 (0)	23* 15–42	84 (47)*	49^ 40–70	8 (100)^	**	**
**Sp10**	6 3–9	1 (8)	6 0–14	19 (11)	19 6–33	2 (25)		
**Np1**	0 0–3	0 (0)	5* 0–10	13 (7)	25^ 18–66	3 (38)	**	**
**Np2**	3 0–6	1 (8)	5 0–10	10 (6)	18^ 9–28	2 (25)	**	
**Np3**	0 0–4	0 (0)	3 0–10	13 (7)	25^ 4–41	3 (38)	**	**
**Np4**	8 1–16	0 (0)	16 6–30	57 (32)*	83^ 35–116	6 (75)	**	**
**Mp1**	0 0–3	0 (0)	0 0–4	1 (0.6)	21^ 13–29	3 (38)^	**	**
**Mp2**	0 0–4	0 (0)	2 0–6	0 (0)	130^ 44–243	7 (88)^	**	**

IL2 responses to individual peptide subpools for the S, N and P proteins were expressed as median and range of responses (spot forming cells/million PBMC, sfc/m), and number and % participants that were positive.

Initial omnibus null hypotheses comparing the magnitude (Kruskal-Wallis) and frequency (Fisher’s Exact) of responses of all groups to each of the subpools were interpreted using an alpha = 0.05. When rejected, pairwise comparisons were performed and interpreted using a Bonferroni-adjusted alpha = 0.0167.

*Significant differences between Group 2 compared to Group 1

**^**significant differences between Group 3 compared to Group 2.

In addition, the magnitudes, and frequencies of responses of each Group were also compared by Kruskall-Wallis (KW) or Chi-Square (Chi-Sq).

**Significant p values indicate that there were significant differences between each group.

**Table 4:** FluoroSpot IFN-γ+IL2 responses to individual S, N and M peptide subpools.

	IFN-γ+IL2							
	GROUP 1 N = 12		GROUP 2 N = 179		GROUP 3 N = 8		KW	Chi-Sq
Peptide Pool	Median Q1-Q3	# Pos (%)	Median Q1-Q3	# Pos (%)	Median Q1-Q3	# Pos (%)	P Mag	P Fre
**Sp1**	0 0–0	0 (0)	0 0–4	3 (2)	5 1–9	1 (13)		
**Sp2**	0 0–0	0 (0)	6* 2–15	19 (11)	19 4–31	3 (38)	**	
**Sp3**	0 0–0	0 (0)	2* 0–6	8 (5)	8 4–14	1 (13)	**	
**Sp4**	0 0–0	0 (0)	3* 0–10	10 (6)	15^ 11–24	2 (25)	**	
**Sp5**	0 0–0	0 (0)	0 0–3	2 (1)	9^ 1–28	2 (25)^	**	**
**Sp6**	0 0–0	0 (0)	0 0–0	0 (0)	0 0–4	1 (13)		**
**Sp7**	0 0–0	0 (0)	10* 3–18	25 (14)	21^ 20–44	3 (38)	**	
**Sp8**	0 0–0	0 (0)	6* 0–12	16 (9)	23^ 13–35	4 (50)^	**	**
**Sp9**	0 0–0	0 (0)	3* 0–8	7 (4)	11^ 9–21	2 (25)	**	**
**Sp10**	0 0–0	1 (8)	0 0–0	1 (0.6)	0 0–6	0 (0)		
**Np1**	0 0–0	0 (0)	0 0–0	0 (0)	8^ 0–19	1 (13)^	**	**
**Np2**	0 0–0	0 (0)	0 0–0	0 (0)	3^ 0–9	0 (0)	**	
**Np3**	0 0–0	0 (0)	0 0–0	0 (0)	8^ 1–9	1 (13)	**	**
**Np4**	0 0–0	0 (0)	2* 0–6	5 (3)	23^ 9–49	4 (50)^	**	**
**Mp1**	0 0–0	0 (0)	0 0–0	0 (0)	3^ 0–8	0 (0)	**	
**Mp2**	0 0–3	0 (0)	0 0–0	0 (0)	50^ 20–73	5 (63)^	**	**

IFN-γ+IL2 responses to individual peptide subpools for the S, N and P proteins were expressed as median and range of responses (spot forming cells/million PBMC, sfc/m), and number and % participants that were positive.

Initial omnibus null hypotheses comparing the magnitude (Kruskal-Wallis) and frequency (Fisher’s Exact) of responses of all groups to each of the subpools were interpreted using an alpha = 0.05. When rejected, pairwise comparisons were performed and interpreted using a Bonferroni-adjusted alpha = 0.0167.

*Significant differences between Group 2 compared to Group 1

**^**Significant differences between Group 3 compared to Group 2.

In addition, the magnitudes, and frequencies of responses of each Group were also compared by Kruskall-Wallis (KW) or Chi-Square (Chi-Sq).

**Significant p values indicate that there were significant differences between each group.

### IFN-γ, IL2, and IFN-γ+IL2 responses to individual S, N, and M peptide subpools

#### Group 1: Uninfected and unvaccinated participants

*S*, *N*, *and M proteins*. The frequencies and median IFN-γ responses (Table 2, Fig 2) were predominantly to the Sp6 (50%, 28 sfc/m) and Mp1 (38%, 22 sfc/m) and were low or absent against other subpools. IL2 responses (Table 3, Fig 2) and IFN-γ+IL2 responses (Table 4, Fig 2) responses were largely low or absent against all subpools, including Sp6 and Mp1.

#### Interpretation

Sp6 contains the S1/S2 subunit cleavage site, and Mp1 contains the M protein transmembrane domains, which may be conserved among HCoVs [22, 36], and may represent prior non-SARS-CoV-2 HCoV infections in these participants.

#### Group 2: Uninfected and vaccinated participants

*S glycoprotein*. The frequencies of IFN-γ responses (Table 2) to each S subpool were higher than Group 1, significantly to Sp2 (54%) and Sp7 (64%); the magnitudes of IFN-γ responses (Table 2, Fig 2) were significantly higher to each S subpool except to Sp6 and Sp10. IFN-γ responses were highest to Sp7 (37 sfc/m), followed in order by Sp6, Sp2, Sp8, Sp9, Sp4, Sp3, Sp1, Sp5 and lowest to Sp10, and appeared to be mostly directed to the S2 subunit (Sp7, Sp8 and Sp9).

The frequencies of IL2 responses (Table 3) were also higher than Group 1, significantly more frequent in seven S subpools, and were also highest to Sp7 (81%); the magnitudes of IL2 responses (Table 3, Fig 2) to eight S subpools were significantly higher, except to Sp5 and Sp10, and were highest to Sp7 (48 sfc/m) followed in order by Sp8, Sp2, Sp9, Sp4, Sp3, Sp1, Sp5, Sp10 and were lowest to Sp6 (3 sfc/m), and like IFN-γ responses were mostly directed to the S2 subunit. The frequencies of IFN-γ+IL2 responses (Table 4) were low and not statistically different than Group 1; the magnitudes of IFN-γ+IL2 responses (Table 4, Fig 2) were also low but significantly higher than Group 1 to six S subpools and were also highest to Sp7 (10 sfc/m), followed in order by Sp2, Sp8, Sp9, Sp4 and Sp3, also mostly to the S2 subunit.

*N protein*. The frequencies of IFN-γ responses (Table 2) were low or absent, except to Np4 (22%, 12 sfc/m, respectively) and were significantly higher than Group 1 (Table 2, Fig 2). IL2 responses (Table 3) to Np4 were also significantly more frequent (32%) and of greater magnitude (16 sfc/m); IL2 responses to Np1 (5 sfc/m) were significantly higher than Group 1 (Table 3, Fig 2). The frequencies of IFN-γ+IL2 responses (Table 4) and magnitude (Table 4, Fig 2) to Np4 (2 sfc/m) were low but also higher than Group 1.

*M protein*. The frequencies (Table 2) and magnitudes (Table 2, Fig 2) of IFN-γ responses (Table 2, Fig 2) to Mp1 (52%, 24 sfc/m) and Mp2 (8%, 0 sfc/m) were like those observed in Group 1. The frequencies and magnitudes of IL2 and IFN-γ+IL2 responses (Table 3, Table 4) were low or absent, as in Group 1.

#### Interpretation

Vaccination-induced IFN-γ, IL2, and IFN-γ+IL2 responses were predominantly of greater frequency and magnitude to subpools aligned with the S2 subunit: Sp7 spans the S2’ cleavage site within the S2 subunit, Sp8 spans the heptad repeat and central helix, and Sp9 that spans part of the connector domain, and lower responses were to Sp2 that spans part of the subunit 1 N-terminal domain. Interestingly, responses to Sp6 were unchanged after vaccination despite being contained in the S-based vaccine. The vaccine lacked N and M proteins, and IFN-γ, IL2, and IFN-γ+IL2 responses to the N protein, especially Np4 that spans the N4 and N5 domains, are consistent with seroconversion to the N protein that may be derived from asymptomatic infection [13]. However, asymptomatic infections did not appear to affect responses to the M protein. Alternatively, these represent conserved sequences shared with conserved sequences from prior HCoV infections or non-specific activation of memory T cells.

#### Group 3: Infected and vaccinated participants

Overall, IFN-γ, IL2, and IFN-γ+IL2 responses to individual S, N, and M peptide subpools are significantly greater than uninfected and vaccinated participants.

*S glycoprotein*. The frequencies of IFN-γ responses (Table 2) in Group 3 were higher than Group 2 to all S subpools except Sp6, although only frequencies to Sp4 (75%), Sp5 (75%) and Sp8 (88%) were significantly higher. All participants had positive IFN-γ responses to Sp7. The magnitudes of IFN-γ responses (Table 2, Fig 2) were also higher to nine S subpools, except to Sp6 that were unchanged, and were significantly higher in five S pools. IFN-γ responses were directed to the S1 and S2 subunits and were highest to Sp4 (71 sfc/m), followed in order by Sp7 (66 sfc/m), Sp2, Sp8, Sp9, Sp5, Sp6, Sp3, Sp1 and lowest to Sp10 (11 sfc/m). IFN-γ responses to Sp5 and Sp4 had the greatest fold-increases (17-times, 5.5-times, respectively) compared to Group 2, suggesting responses increased more to the S1 subunit compared to Group 1.

The frequencies of IL2 responses (Table 3) to all subpools were all higher but were only significantly higher to Sp5 (63%) and Sp9 (100%); all participants had IL2 responses to Sp7, Sp8 and Sp9. Magnitudes of IL2 responses (Table 3, Fig 2) were higher to all S subpools, except Sp6, and were significantly higher to Sp5 (41 sfc/m), Sp7 (70 sfc/m) and Sp9 (49 sfc/m). IL2 responses were also directed to the S1 and S2 subunits and were highest to Sp2 (76 sfc/m), followed in order by Sp7, Sp8, Sp9, Sp4, Sp5, Sp3, Sp1, Sp10 and lowest to Sp6 (6 sfc/m). IL2 responses to Sp5 had the highest fold increase (5.2-times).

The frequencies of IFN-γ+IL2 responses (Table 4) were higher than Group 2 to all S subpools except Sp10 and were significantly higher to Sp5 (25%) and Sp8 (50%). Magnitudes of IFN-γ+IL2 responses (Table 4, Fig 2) to all S subpools except Sp6 and Sp10 were also higher than Group 2, and this was significant to Sp4, Sp5, Sp7, Sp8 and Sp9. IFN-γ+IL2 responses were directed to the S1 and S2 subunits and were highest to Sp8 (23 sfc/m), followed by in order Sp7 (21 sfc/m), Sp2, Sp4, Sp9, Sp5, Sp3, Sp1, and absent to Sp6 and Sp10.

#### Interpretation

Prior infection with SARS-CoV-2 broadened responses to the S1 and S2 subunits as subpools Sp2 and Sp4 are within the S1 subunit spanning the N-terminal domain and the RBD domain, and Sp7, Sp8 and Sp9 are within the S2 subunit spanning the fusion peptide and the S2’ cleavage site, the HR2 heptad repeat, the central helix and connector domain. Therefore, we conclude that prior SARS-CoV-2 infection before vaccination broadened responses to the S1 and S2 subunits and may contribute to greater vaccine efficacy [10, 11]. However, we did not measure responses of Group 3 after infection and prior to vaccination and we cannot distinguish the relative contributions of infection and vaccination.

*N protein*. The frequencies of IFN-γ responses (Table 2) to three of four N subpools were significantly higher than Group 2 to Np1 (38%), Np3 (38%) and Np4 (88%). Magnitudes of IFN-γ responses (Table 2, Fig 2) were significantly higher to all four subpools and were highest to Np4 (98 sfc/m), Np3 (19 sfc/m), Np2 (18 sfc/m) and were lowest to Np1 (14 sfc/m).

The frequencies of IL2 responses (Table 3) were like levels found in Group 2, but magnitudes to all four N subpools (Table 3, Fig 2) were significantly higher than Group 2, and were highest to Np4 (83 sfc/m) followed by Np3 (25 sfc/m), Np1 (25 sfc/m) and lowest to Np2 (18 sfc/m).

The frequencies of IFN-γ+IL2 responses (Table 4, Fig 2) were significantly higher than Group 2 to Np4 (50%) and Np1 (13%). Magnitudes of responses (Table 4, Fig 2) were significantly higher than Group 2 to each N subpool and were highest to Np4 (23 sfc/m), Np3 (8 sfc/m), Np1 (8 sfc/m) and lowest to Np2 (3 sfc/m).

#### Interpretation

prior SARS-CoV-2 infection broadened IFN-γ, IL2, and IFN-γ+IL2 responses after vaccination particularly to Np4 that aligns with part of the N4/CTD domain and the N5 domain and had less effect on responses to Np3 that also aligns with the N4/CTD domain and to Np1 and Np2 that align with the N1 and N2/NTD domains. Since the vaccine only contains the S glycoprotein, we suggest that the broadened and increased responses to the N protein are probably derived from the prior SARS-CoV-2 infection, or possible cross-reactive sequences in Np4 shared with other HCoVs as we found responses to Np4 in uninfected and unvaccinated participants.

*M protein*. The frequencies (Table 2) and magnitudes (Table 2, Fig 2) of IFN-γ responses (Table 2, Fig 2) were significantly higher than Group 2 to Mp2 (100%, 108 sfc/m), and were the highest magnitude of IFN-γ responses to any S, N or M subpool; responses to Mp1 were unchanged compared to Group 2 (88%, 29 sfc/m). The frequencies and magnitudes of IL2 (Table 3, Fig 2) and IFN-γ+IL2 (Table 4, Fig 2) responses were significantly higher than Group 2 to the Mp2 subpool (88%, 130 sfc/m; 63%, 50 sfc/m, respectively), and were also the highest magnitude of IL2 and IFN-γ+IL2 responses to any S, N or M subpool, IL2 responses were unchanged to Mp1 (38%, 21 sfc/m) and IFN-γ+IL2 responses were absent to Mp1.

#### Interpretation

Prior SARS-CoV-2 infection broadened IFN-γ, IL2 and IFN-γ+IL2 responses after vaccination to include Mp2 and were the highest responses of all subpools, whereas responses to Mp1 were not different compared to other groups. The M protein residues 1–135 transmembrane domains align with Mp1 and are proposed to interact with the S glycoprotein, and may therefore be conserved, whereas Mp2 aligns with the CTD that remains in the cytosol and may play a role in SARS-CoV assembly [37]. As with broadened responses to the N protein, we suggest that the broadened responses to the M protein are probably derived from the prior SARS-CoV-2 infection.

Since we detected IFN-γ responses to Sp6 and Mp1 in uninfected unvaccinated participants, as well as in uninfected participants after vaccination, we suggest that they were derived from HCoV infections containing cross-reactive sequences with SARS-CoV-2. Consistent with that origin, we found that IFN-γ responses to Sp6 and Mp1 were highly correlated with each other in each group (S3 Fig in S1 File). We only detected IFN-γ responses to Sp7 and Sp8 after vaccination and not in uninfected unvaccinated participants, suggesting these were derived from the S-based vaccine, and these were also highly correlated with each other in vaccinated Group 2 and Group 3 participants, but not in uninfected unvaccinated participants, consistent with origin of these responses from vaccination (S3 Fig in S1 File).

These significant differences between frequencies, magnitudes and protein-specific responses in each group were also consistent with Kruskall-Wallis analyses of magnitudes and Chi-Square analyses of frequencies of these responses across the three groups (Tables 2–4). These analyses further confirmed that responses to Sp6 and Mp1 remained unchanged in uninfected and unvaccinated participants and after vaccination of participants with or without prior SARS-CoV-2 infection.

### Correlation of FluoroSpot and IgG antibody responses

**Fig 3:**
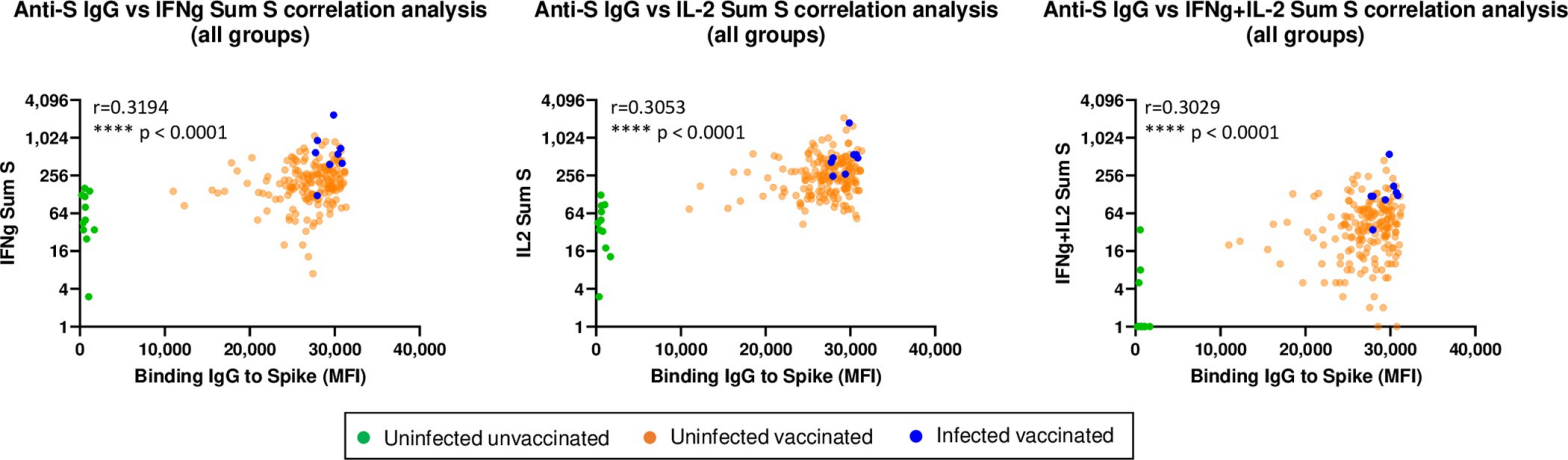
Correlations between summed IFN-γ, IL2, and IFN-γ+IL2 responses to S sub-pools and serum IgG responses to Spike glycoprotein. Summed IFN-γ, IL2, and IFN-γ+IL-2 responses (spot forming cells/million PBMCs, sfc/m) to S glycoprotein correlated with serum IgG responses to Spike glycoprotein. Total n per graph = 198: Uninfected unvaccinated n = 11, Uninfected vaccinated n = 179, Infected vaccinated n = 8. Of note, 8 individuals in the uninfected unvaccinated group and 2 individuals in the uninfected vaccinated group had values of zero for frequencies of cells producing both IFN-γ and IL-2 in response to S peptides. While the zero values were used for statistical analyses, they were plotted as values of 1 to enable visualization on the log axis. Correlations were assessed using the non-parametric Spearman’s correlation analysis.

Frequencies of Spike peptide-specific cellular responses correlated with serum levels of S-specific IgG antibodies drawn at the same time as the PBMCs used for cellular response assays. As seen in Fig 3, anti-S IgG antibody responses correlated moderately with IFN-γ summed S, summed IL-2 S, and summed IFN-γ+IL-2 S responses with high degrees of significance (p values < 0.0001). Frequencies of IFN-γ producing cells and IL-2 producing cells exhibited similar correlations with antibody levels (rho values of 0.3194 and 0.3053), and strength of correlation was not improved when analyzing cells producing both IFN-γ and IL-2 (rho 0.3029).

### Hierarchies of responses to S, N and M proteins

We next used Principal Component Analysis (PCA) to transform the complex primary immune response data to identify trends and patterns within that data.

**Fig 4:**
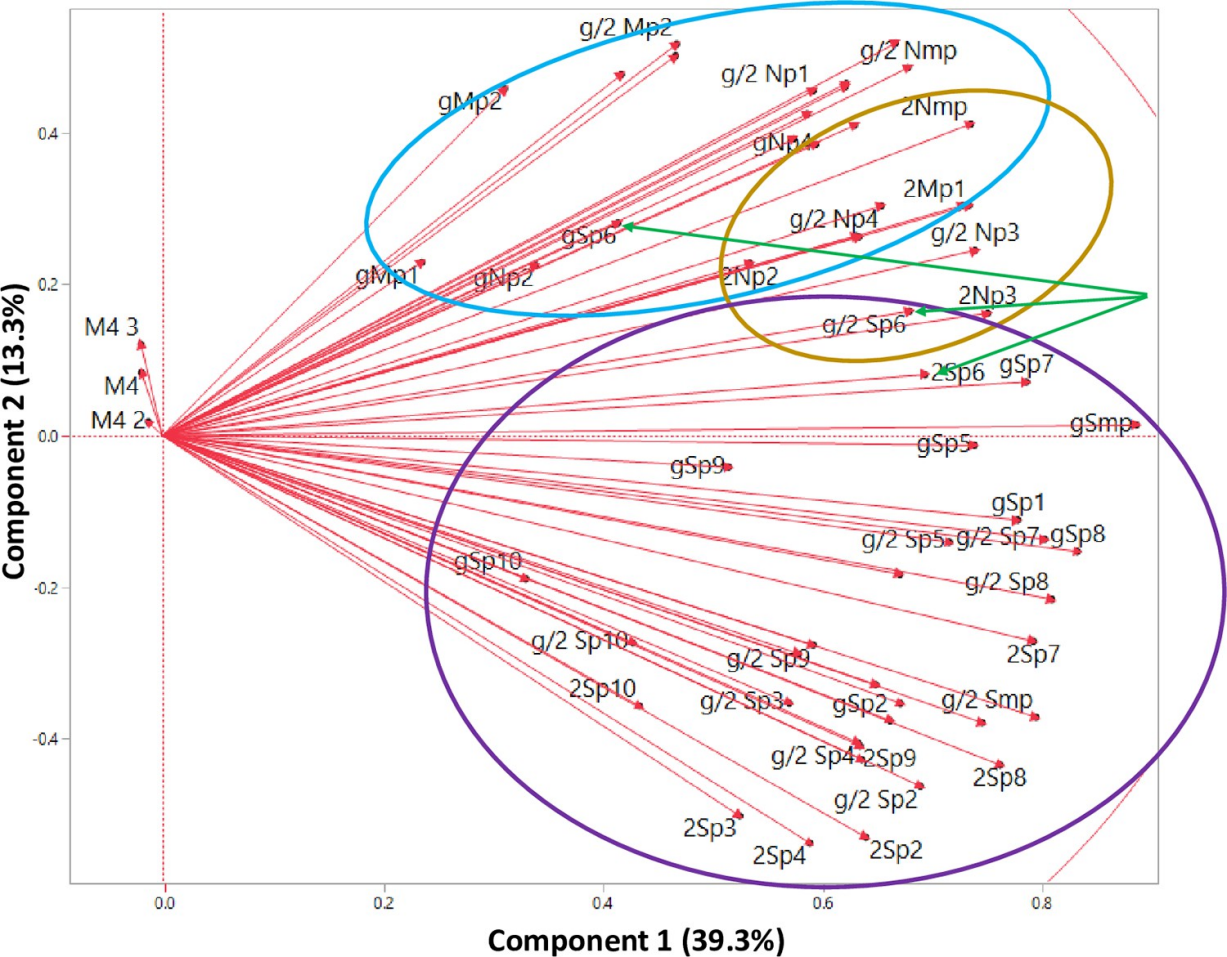
Hierarchical distribution of IFN-γ, IL2, and IFN-γ+IL2 responses to S, N, and M peptide subpools. Responses to each stimulant were used, including to all 10 S subpools, all 4 N subpools and each M subpool. In addition, responses to megapools each containing all S, or N, or M peptides combined into one pool, Smp, Nnp, and Mmp respectively, were also used. IFN-γ responses (g), IL2 responses (2), IFN-γ+IL2 responses (g/2). When Components 1 and 2 are compared, responses to Medium only (M4, M4 2 and M4 3) fell outside this correlation. However, S and M peptides fall within distinct aggregate areas defined by purple or blue circles respectively, although responses to N protein (gold circle) overlap the S and M protein areas. S glycoprotein peptide subpool Sp6 falls within each aggregated area (green arrows): IFN-γ (gSp6), IL2 (2Sp6), IFN-γ+IL2 (g/2Sp6).

All immune response variables that had a Pearson r ≥0.3 with at least one other parameter were included in the PCA. Data sets were appropriate for PCA with a KMO value of 0.90 and a significant result (p<0.0001) in the Bartlett test for sphericity. Principal components 1 and 2 had eigen values ≥1 and together explained 81.3% of the variability in immune responses across the study groups. Components1 and 2 accounted for 39.3% and 13.3% of the total variability, respectively (Fig 4). All peptide subpools and megapools (other than medium controls M4, M2, M3) immune response variables were positively correlated with Component 1 (Fig 3). Interestingly, variables were spread across Component 2 generally grouping into responses to the N, S and M peptide subpools, and megapools with the S glycoprotein peptide subpool Sp6 spread across the other subpools perhaps reflecting that it contains a conserved sequence shared among different HCoVs.

**Fig 5:**
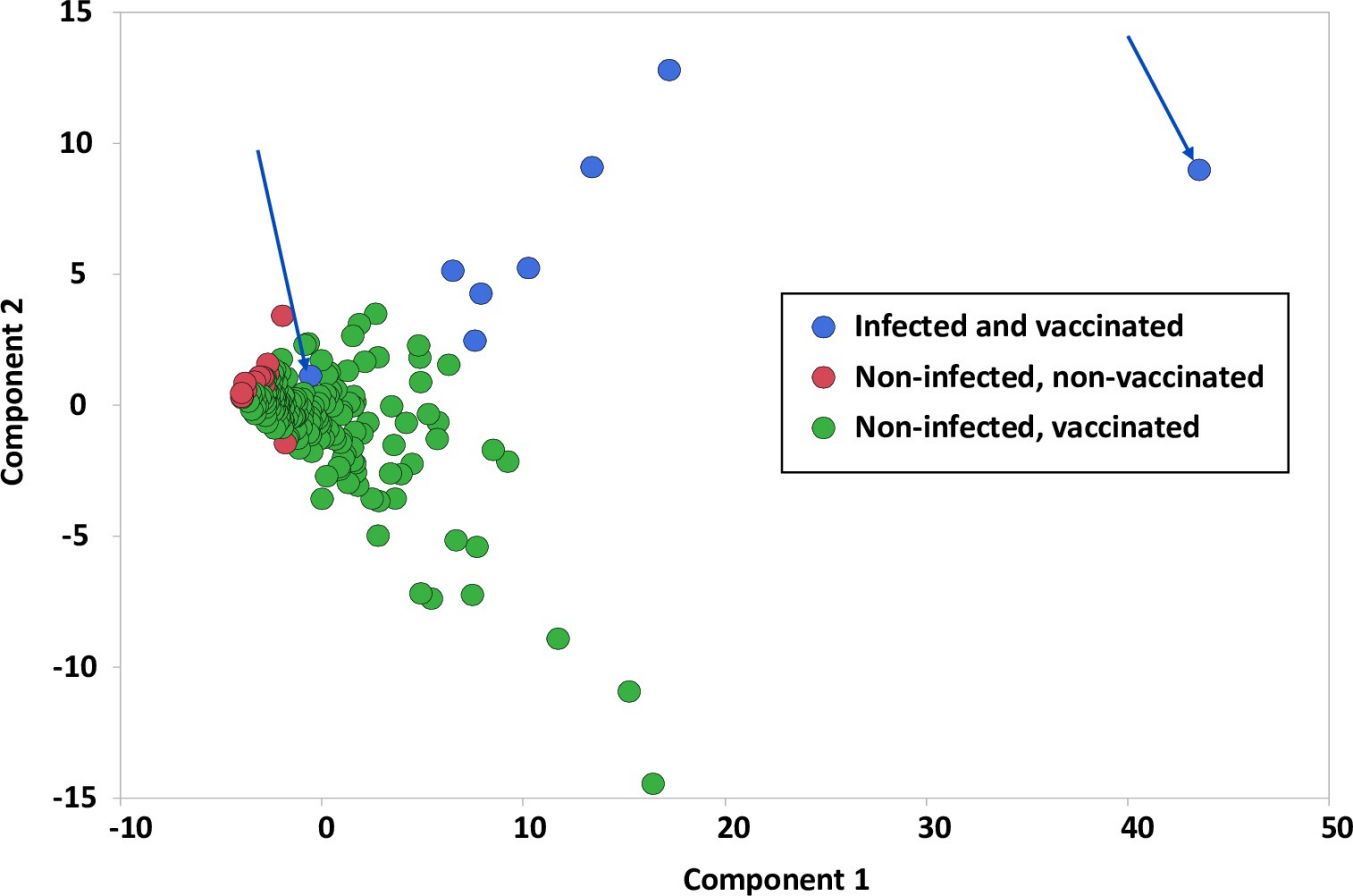
Scatter plot comparison of all subjects. Component 1 and Component 2 values appear to differentiate participants based on prior infection and vaccination. Participants who were infected and vaccinated had higher Component 2 values reflecting responses to the N and M proteins; however, there are two outlier subjects (blue arrows). In contrast, uninfected and vaccinated participants had lower Component 2 values reflecting the responses to the S protein. Participants who were uninfected and unvaccinated are distinct and cluster around 0.

The principal component analysis can be thought of as a variable reduction technique whereby new variables (Component 1 and Component 2) are created to explain the majority of the heterogeneity in the data. These two components are then analyzed to elucidate differences between groups. Component 1 and Component 2 values for immune responses to the subpools and megapools appeared to group together based on prior infection and vaccination with SARS-CoV-2 vaccines. When Component 1 and Component 2 were graphed on a scatter plot (Fig 5), participants in each of the three study groups appeared to group together, and each was distinct from the other two groups. Uninfected and unvaccinated participants had a negative Component 1 and a Component 2 value of approximately zero. Uninfected and vaccinated participants had lower Component 2 and higher Component 1 values generally reflecting more robust responses to the S subpools and S megapool. In contrast, infected and vaccinated participants had higher Component 2 and higher Component 1 values generally reflective of more robust responses to the N and M subpools; two participants (blue arrows) had responses that differed from the rest of this group; of these, one subject with high Component 1 values had the highest IFN-γ, IL2 and IFN-γ+IL2 responses to each N subpool; the second subject had low IFN-γ, IL2 and IFN-γ+IL2 responses to N and M proteins peptide subpools and grouped with uninfected and vaccinated participants who also had low responses to N and M proteins.

### Heat map of Pearson correlations between IFN-γ, IL2 and IFN-γ+IL2 responses with each S, N, and M peptide subpool

**Fig 6:**
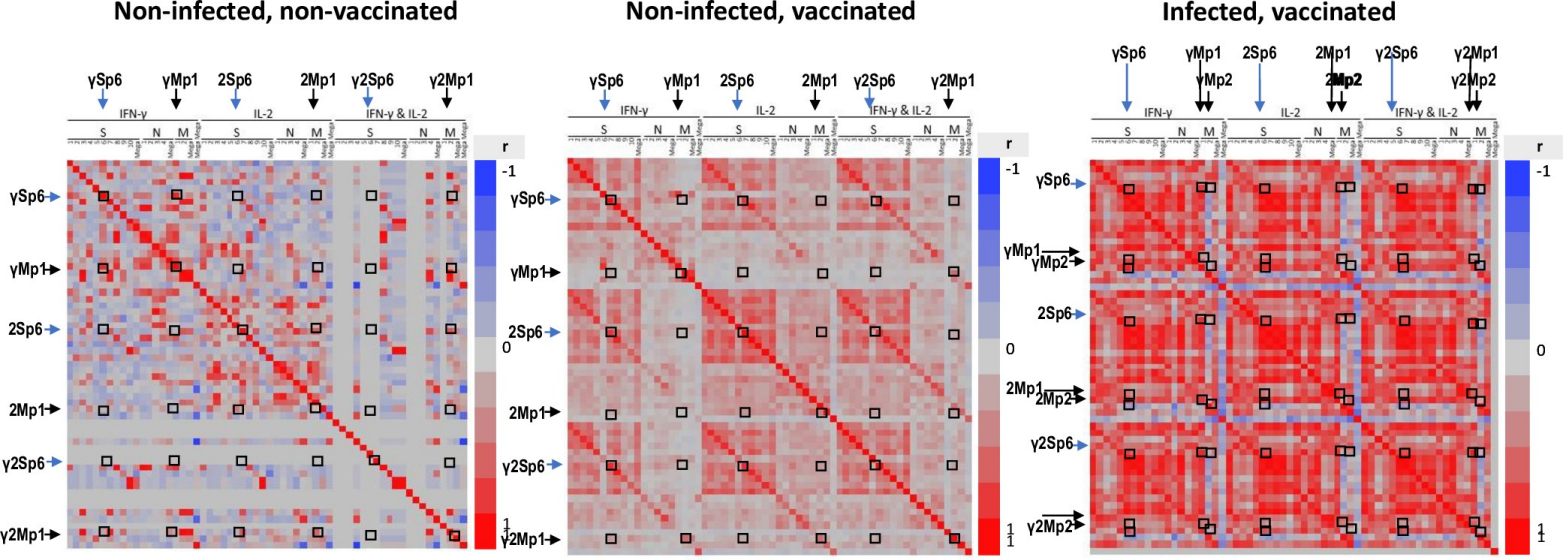
Heat map profiles of all IFN-γ, IL2, and IFN-γ+IL2 responses to each S, N, and M peptide subpool. Group 1 = uninfected, unvaccinated; Group 2: uninfected, vaccinated; Group 3: infected, vaccinated. All IFN-γ (γ), IL2 (2), and IFN-γ+IL2 (γ2) responses of individual subjects with each S, N and M peptide subpool were compared with each other using the Pearson correlation coefficient (r), a value of “1” is shown in bright red and represents 100% positive correlation, and a value of -1 in bright blue represents 100% negative correlation. Intersection of each parameter is boxed. Sp6 and Mp1: γSp6 and γMp1, 2Sp6 and 2Mp1, and γ2Sp6 and γ2Mp1, were mapped on Groups 1, 2, and 3. Sp6 and Mp2: γSp6 and γMp2, 2Sp6 and 2Mp2, and γ2Sp6 and γ2Mp2, were mapped on Group 3.

We next evaluated the correlation of immune response parameters using heat maps of the Pearson correlation coefficients (Fig 6). IFN-γ, IL2, and IFN-γ+IL2 responses to Sp6 and Mp1 are significantly correlated in uninfected and unvaccinated participants and less so in uninfected and vaccinated participants or infected and vaccinated participants, representing their possible common origin from other HCoV infections, and consistent with the correlation in S3 Fig in S1 File. However, IFN-γ, IL2 and IFN-γ+IL2 responses to Mp2 are not correlated with Sp6 in infected and vaccinated participants (S3 Fig in S1 File) and may reflect the high level of responses to Mp2 seen in participants who were infected prior to vaccination. These maps will allow mining of other correlations between peptide subpools, for example IFN-γ responses to Sp7 and Sp8 were highly correlated in the vaccinated with or without prior infection groups, but not in the uninfected and unvaccinated group (S3 Fig in S1 File), and further support the distinctness of the three groups as shown in Fig 4 and Tables 1–4.

## Discussion

We made three important findings in these studies that are highly relevant to our understanding of immune responses to SARS-CoV-2 infections and the efficacy of current SARS-CoV-2 vaccines. **(1)** After vaccination of participants with no prior SARS-CoV-2 infection, using the S glycoprotein-based mRNA vaccines, IFN-γ and IL2 responses to the S glycoprotein were present in almost all participants, IFN-γ+IL2 responses were low, and responses were predominantly directed to the S glycoprotein S2 subunit, and these responses correlated with our previously reported [13] IgG responses to the S glycoprotein.

Response to one subpool of the N protein (Np4), and to one M protein subpool (Mp1) were also detected in some participants. **(2)** After vaccination of participants with a prior confirmed SARS-CoV-2 infection, IFN-γ, IL2, and IFN-γ+IL2 responses were significantly higher than vaccinated participants without previous infection and were more broadly directed to the S glycoprotein S1 and S2 subunits, as well as N and M protein subpools. **(3)** Participants without prior infection and who were not vaccinated had IFN-γ responses to one S subpool (Sp6) and one M subpool (Mp1) that may represent cross-reactive epitopes from previous infection with antigenically-related HCoVs, and specific responses did not increase after vaccination.

Our findings that previously SARS-CoV-2 infected participants had broader and significantly higher FluoroSpot IFN-γ, IL2 and IFN-γ+IL2 responses to the S, M and N proteins than vaccination of uninfected participants agree with others who have also shown that participants recovered from SARS-CoV-2 infection develop robust immune responses after mRNA vaccines [38–40]. The vaccine is based on the S glycoprotein, and it is likely that responses to the N and M proteins were derived from the prior SARS-CoV-2 infection, or possibly previous infection with other HCoVs. However, other studies have demonstrated T cell responses to the N and M proteins after vaccination of convalescent individuals [35, 41, 42], suggesting activation of memory T cells after an antigenic stimulus [35]. Our results are also consistent with a similar study that also used ELISpot IFN-γ assays and S glycoprotein peptide subpools to measure responses of fresh PBMCs from HCWs who received a single or two doses of the Pfizer-BioNTech BNT162b vaccine and were either previously infected or uninfected with SARS-CoV-2 [43]; after vaccination, summed S-specific ELISpot IFN-γ responses were significantly higher in previously infected HCWs than in infection-naïve participants, and responses were distributed among the four peptide subpools that spanned the S1 and S2 subunits. After two BNT162b2 doses in SARS-CoV-2- naive participants, total S-specific T cell responses (S1 and S2 subunit responses) were equivalent to those after a single dose in previously infected individuals [43]. Thus, our studies add to findings that prior SARS-CoV-2 infection broadens immune responses to the S glycoprotein after vaccination, as well as the N and M proteins.

In other studies, S glycoprotein-specific T cell responses were also greater after vaccination in previously SARS-CoV-2-infected participants than those who were infection-naïve [44, 45], and it is possible that vaccination boosted CD4+ T cell responses to the S glycoprotein that were below the threshold of detection [46, 47]. CD8+ T cell responses might be directed to non-S glycoproteins following natural infection, but in infection-naïve participants CD8+ T cell responses are focused on the S glycoprotein contained in the vaccine [43]. Prior infection also broadens responses of the As26.Cov2.2 immune responses [48] and may induce more persistent nasopharynx-homing SARS-CoV-2-specific T cells [49]. Recent studies suggest the necessity of COVID-19 vaccination in participants who have had a prior COVID-19 infection [50], and these studies complement the importance of hybrid immunity that was shown in a study of hospitalizations that vaccination protects against reinfection and hospitalization, especially against the Delta variant [11].

The emergence of SARS-CoV-2 variants of concern (VOC) [51] include Omicron which has 14 non-synonymous mutations in the S glycoprotein [52] in three clusters within the RBD and HR1 domains [53]. These clusters align with Sp3, Sp4 (two clusters), and Sp7/Sp8 (one cluster), In addition, Omicron has four changes in the N protein that align with Np1 and Np2, including the P13L located in a characterized T cell epitope aligned with Np1 [54, 55]. Additionally, there are three changes in the M protein aligned with Mp1 [54]. Current evidence suggests that in participants immunized with the mRNA- or adenovirus-based COVID-19 vaccines, up to 90% of T cell responses to the Omicron variant were preserved against Omicron [56]. Thus, it is likely that our observed responses will be functionally effective against multiple VOCs. Our future planned studies will fine map immunodominant epitopes within the S, N and M proteins, and determine if Omicron mutations affect IFN-γ responses, particularly since the S-based vaccine elicited immune responses to particularly to Sp4, Sp7 and Sp8.

We are also interested in identifying cross-reactive epitopes shared by SARS-CoV-2 and other common HCoV, as there is accumulating evidence that suggests that SARS-CoV-2 infection is often milder or asymptomatic in children and young adults [57]. Children mount robust antibody responses compared to adults, with pre-existing immunity cross derived from prior HCoV exposures [58, 59], and cross-reactive antibodies were largely directed to the S glycoprotein [60]. Cross-reactive ELISpot IFN-γ responses in children and young adults are also directed to the S glycoprotein, (particularly the S2 subunit) but not the N and M proteins [7, 59, 61, 62]. We also found FluoroSpot responses in uninfected unvaccinated participants to Sp6 that spans part of the S2 subunit. Longitudinal studies suggest that HCoVs induce a stable pool of memory CD4+ T cells that may account for the FluoroSpot IFN-γ responses in participants with no prior COVID-19 infection [63]. Cross-reactive epitopes present in SARS-CoV-2 and other HCoVs have been identified in the S, N and M proteins [46, 61, 64–66], including within the Sp6 and Mp1 subpools.

Remarkably, though, frequencies of IFN-γ producing cells against the Sp6 and Mp1 subpools were not substantially greater in infected plus vaccinated individuals compared to the other two groups, potentially suggesting that previous T cell immunity to these regions impairs boosting to these regions in response to SARS-CoV-2 or infection, and, in the case of Sp6, after S-based vaccination. Further work is needed to determine whether this is related to the concept of “original antigenic sin”, which suggests that responses to a primary infection can significantly influence responses to the next strain encountered [67–69] or other factors. Fine mapping of specific responses to Sp6 and Mp1 15mers is required to establish the relationship between Sp6 and Mp1 with other HCoV proteins, and whether these represent conserved epitopes that that could be investigated for use in biomarkers of immunity studies.

Finally, using PCA, we found that each of these three groups of participants aggregated across Component 1 and Component 2 based on responses to S, N or M proteins and that prior SARS-CoV-2 infection elicits responses to the N and M proteins that drives the vaccine-induced responses differently than vaccination in the absence of prior infection. Recent studies demonstrated increased protection in persons with previous infection, with or without vaccination, compared to vaccination alone [10, 11]. We suggest that vaccination of participants with prior SARS-CoV-2 infection induced significantly higher FluoroSpot IFN-γ responses and may also confer higher protection to SARS-CoV-2 infection.

### Limitations

There are several limitations to this study. Firstly, the study population is HCWs recruited in areas of the WRNMMC with a high frequency of contact with confirmed or suspected cases of COVID-19 patients and may acquire a viral load that may not be reflective of the general population, limiting the generalizability of the findings. Although we evaluated immune responses to the S, N and M proteins, it is likely that responses to other proteins may also have a significant role in immunity to SARS-CoV-2. We only measured IFN-γ and IL2 producing cells and it is likely that phenotypic analyses may identify the roles of CD4+ and CD8+ T cell responses. Finally, this study was heavily weighted to uninfected vaccine-immunized participants, and numbers of participants that were infected and vaccine-immunized, or uninfected and unimmunized were much lower; although differences between these groups were statistically significant, these outcomes might be either confirmed or differ in more balanced groups.

## Supporting information

S1 File (DOCX)
