# Supplementary material for: Cellular interferon-gamma and interleukin-2 responses to SARS-CoV-2 structural proteins are broader and higher in those vaccinated after SARS-CoV-2 infection compared to vaccinees without prior SARS-CoV-2 infection
Source: PLoS One. 2022 Oct 17;17(10):e0276241. doi: 10.1371/journal.pone.0276241 (PMC9576055; doi:10.1371/journal.pone.0276241)
Supplement: S1 File — (DOCX) [file pone.0276241.s001.docx]

**Cellular interferon-gamma and interleukin-2 responses to SARS-CoV-2 structural proteins are broader and higher in those vaccinated after SARS-CoV-2 infection compared to vaccinees without prior SARS-CoV-2 infection**

**Supplementary Information**

**Figure S1. FluoroSpot IFN-γ, IL2, and IFN-γ+IL2 spot forming patterns to S glycoprotein Sp7 subpool compared to unstimulated controls**

**
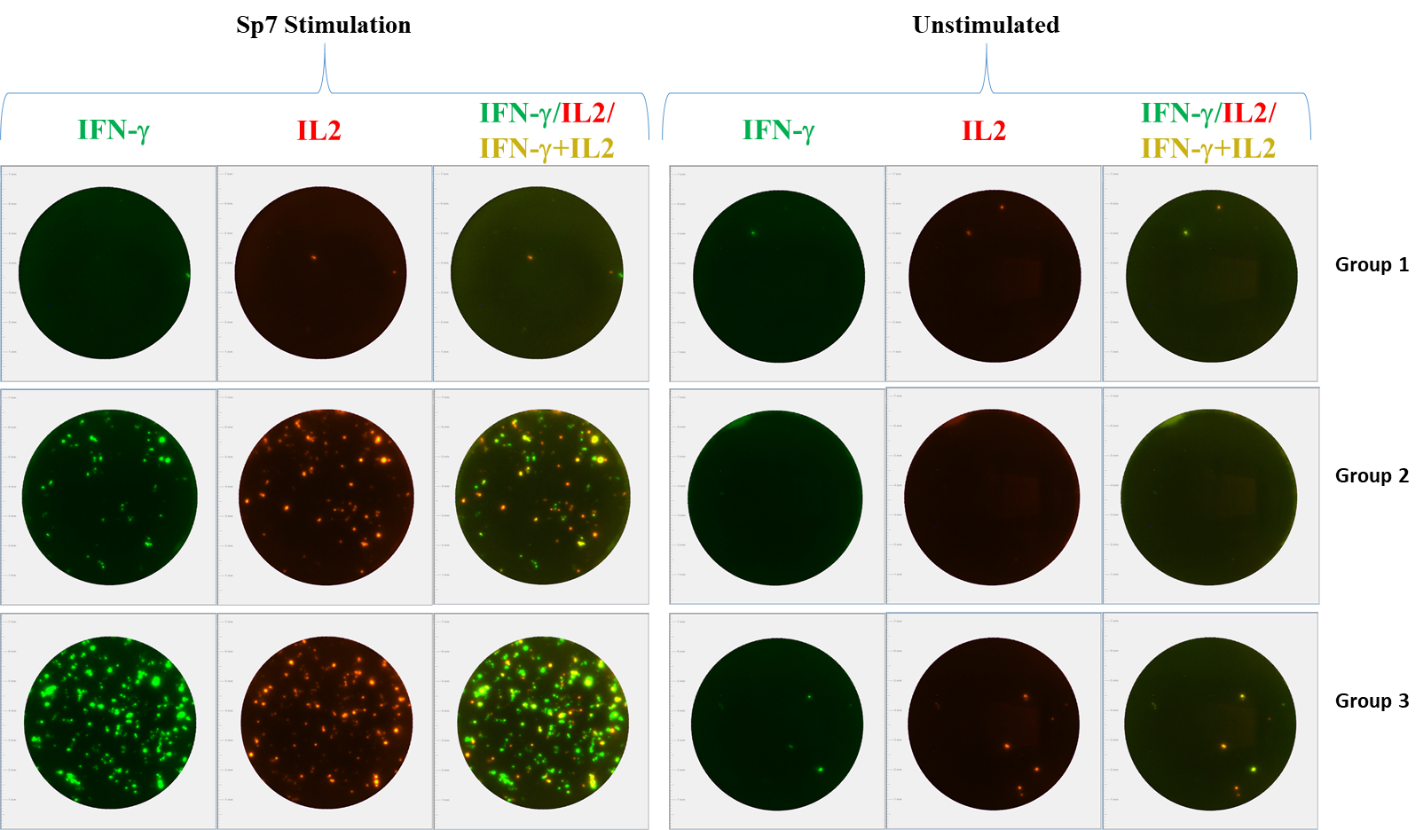
**

PBMC from one representative subjects from each of the three study Groups: **Group 1,** uninfected, unvaccinated; **Group 2,** uninfected, vaccinated, and **Group 3**, Infected, vaccinated subjects were stimulated with S glycoprotein peptide subpool Sp7 or were unstimulated. IFN-γ (green), IL2 (red), or IFN-γ+IL2 (yellow) responses after Sp7 were distinct from unstimulated responses.

**Figure S2: Relationship of summed IFN-γ. IL2, and IFN-γ+IL2 responses to S, N and M proteins, and time after vaccination**

Regression analysis of IFN-γ, IL2 and IFN-γ+IL2 responses to the S glycoprotein, and N and M proteins, and time after vaccination in Group 2 show no correlation. There were also no correlations between IFN-γ, IL2 or IFN-γ+IL2 responses and time after vaccination. These indicate that responses were maintained unchanged for up to 155 days.

**Figure S3: regression analysis of IFN-γ. IL2, and IFN-γ+IL2 responses to Sp6 and Mp1, and Sp7 and Sp8 subpools**

Among each group, correlations between FluoroSpot IFN-γ responses to Sp6 and Mp1, and between Sp7 and Sp8, were calculated using Pearson’s coefficient where outcomes were significant when p=<0.05.
